# Combination of Carbonate of Iron with Sulphate of Quinine in Remittent Fever

**Published:** 1847-01

**Authors:** 


*Combination of Carbonate of Iron with Sulphate of Quinine in Remittent Fever.*—Prof. Lippich, of Padua, recommends the addition of the carbonate of iron to the sulphate of quinine in the treatment of periodical fevers. The following is his formula: ℞. Carbonate of iron, one gramme; sulphate of quinine, one gramme; extract of taraxacum, q. s. To be made into a mass of proper consistency, and divided into thirty pills, two of which are to be taken every two hours. The carbonate of iron may be gradually increased to two grammes.—*Ibid.*

